# ADAPTATION OF GAMEPLAN4CARE FOR HOSPITAL CARE AND CARE TRANSITIONS

**DOI:** 10.1093/geroni/igae098.2038

**Published:** 2024-12-31

**Authors:** Molly Horstman, Tracy Evans, Crystal Guo, Mandi Sonnenfeld, Aanand Naik, Alan Stevens, Mark Kunik

**Affiliations:** Baylor College of Medicine, Houston, Texas, United States; Baylor College of Medicine, Houston, Texas, United States; Texas Tech University Health Sciences Center El Paso, El Paso, Texas, United States; Michael E. DeBakey Veterans Affairs Medical Center, Houston, Texas, United States; The University of Texas Health Science Center, Houston, Texas, United States; Baylor Scott & White Health, Belton, Texas, United States; Baylor College of Medicine, Houston, Texas, United States

## Abstract

Adults with dementia rely on family caregivers after hospital discharge. Increased caregiver stress following discharge may lead to worse outcomes for caregivers and adults with dementia. Our aim was to adapt GamePlan4Care, a community-based caregiver support intervention, to support caregivers of hospitalized adults with dementia. GamePlan4Care combines online, self-directed skills training with four phone calls from a dementia care specialist. Potential adaptations were identified through a literature review and semi-structured interviews with health professionals and caregivers. Adaptation prototypes were presented to caregiving research consultants, a stakeholder panel with a caregiver and health professionals, and individuals who implemented GamePlan4Care in the community. All adaptations were reviewed with the GamePlane4Care developer. We used the Framework for Modification and Adaptations-Expanded (FRAME) to describe the adaptations. Initial adaptations were planned and made during the pre-implementation stage. Adaptations included: 1) adding training content for care transitions (medication review, clinical red flags, delirium, functional decline, and medical tasks), 2) modifying the intervention content to improve caregiver engagement, and 3) modifying the context of intervention delivery. Additional adaptations will be made based on caregiver feedback from an ongoing feasibility and acceptability pilot. The recruitment target of 30 caregivers should be reached in June 2024. Preliminary results will be presented at the symposium. Evidence-based interventions designed for outpatient and community settings need to be adapted before they can be implemented as part of hospital care. Using insights from multiple sources, we developed HospitalGamePlan4Care, a new intervention that combines care transitions training with an evidence-based dementia caregiver support intervention.

